# Cascading renal injury after brain death: Unveiling glycocalyx alteration and the potential protective role of tacrolimus

**DOI:** 10.3389/fcell.2024.1449209

**Published:** 2024-08-06

**Authors:** Kaoutar Idouz, Asmae Belhaj, Benoit Rondelet, Laurence Dewachter, Bruno Flamion, Nathalie Kirschvink, Sophie Dogné

**Affiliations:** ^1^ Molecular Physiology Research Unit (URPhyM), Namur Research Institute for Life Sciences (NARILIS), University of Namur (Unamur), Namur, Belgium; ^2^ Department of Cardio-Vascular, Thoracic Surgery and Lung Transplantation, CHU UCL Namur, UCLouvain, Yvoir, Belgium; ^3^ Laboratory of Physiology and Pharmacology, Université Libre de Bruxelles, Brussels, Belgium; ^4^ Clinical Development, Idorsia Pharmaceuticals Ltd., Allschwil, Switzerland

**Keywords:** brain death, kidney, glycocalyx, tacrolimus, endothelial dysfunction

## Abstract

Brain death (BD) is a complex medical state that triggers systemic disturbances and a cascade of pathophysiological processes. This condition significantly impairs both kidney function and structural integrity, thereby presenting considerable challenges to graft viability and the long-term success of transplantation endeavors. Tacrolimus (FK506), an immunosuppressive drug, was used in this study to assess its impact as a pretreatment on brain death-induced renal injury. This study aimed to investigate changes associated with brain death-induced renal injury in a 4-month-old female porcine model. The experimental groups included brain death placebo-pretreated (BD; n = 9), brain death tacrolimus-pretreated using the clinical dose of 0.25 mg/kg the day before surgery, followed by 0.05 mg/kg/day 1 hour before the procedure (BD + FK506; n = 8), and control (ctrl, n = 7) piglets, which did not undergo brain death induction. Furthermore, we aimed to assess the effect of FK506 on these renal alterations through graft preconditioning. We hypothesized that immunosuppressive properties of FK506 reduce tissue inflammation and preserve the glycocalyx. Our findings revealed a series of interconnected events triggered by BD, leading to a deterioration of renal function and increased proteinuria, increased apoptosis in the vessels, glomeruli and tubules, significant leukocyte infiltration into renal tissue, and degradation of the glycocalyx in comparison with ctrl group. Importantly, treatment with FK506 demonstrated significant efficacy in attenuating these adverse effects. FK506 helped reduce apoptosis, maintain glycocalyx integrity, regulate neutrophil infiltration, and mitigate renal injury following BD. This study offers new insights into the pathophysiology of BD-induced renal injury, emphasizing the potential of FK506 pretreatment as a promising therapeutic intervention for organ preservation, through maintaining endothelial function with the additional benefit of limiting the risk of rejection.

## 1 Introduction

The scarcity of available kidney transplants, coupled with high demand, often requires accepting donor kidneys with broad eligibility criteria. In organ transplantations, most available organs come from brain-dead donors. However, brain death (BD), and the neuroendocrine reflexes that accompany it, induce deleterious effects on the graft and compromise its outcome in the recipient. Cadaveric transplants have poorer graft survival than those originating from living related donors (Terasaki et al., 1995; Hariharan et al., 2000). Despite these challenges, kidney transplantation from brain-dead donors remains the main treatment for patients with end-stage renal disease, providing improved quality of life and long-term survival (Kerforne et al., 2019).

The pathophysiology of BD–induced organ injury involves a cascade of events including sympathetic, immunologic, and inflammatory responses. Serum levels of various inflammatory markers, such as TNF-α, interleukin (IL)-6, IL-8, pro-inflammatory IL-6–to–IL-10 ratio, and monocyte chemoattractant protein-1 (MCP-1), are significantly increased after BD, reflecting a systemic inflammatory response (Schwarz et al., 2018; Weaver et al., 2018; Belhaj et al., 2022). In turn, this systemic inflammation leads to graft dysfunction and sometimes poor graft outcomes following transplantation (Avlonitis et al., 2003). Lung transplants are less affected than heart transplants, renal transplant outcomes are notably compromised by cerebrovascular causes of BD (Watts et al., 2013).

As the kidneys receive around 20% of cardiac output and filter up to 150 mL of plasma per minute, they are inherently highly exposed to inflammatory mediators released during BD (Gomez et al., 2014; Jedlicka et al., 2020). Clinical studies have revealed that BD affects both renal function and morphology, and that tubular damage, interstitial inflammation, increased apoptosis and endothelial activation are observed (Eijnden et al., 2003; Rebolledo et al., 2016; Civiletti et al., 2019; Jager et al., 2019). Activation of NF-kB in the kidney is also reported, increasing local inflammation (Bouma et al., 2009). Increased expression of cytokines, chemokines, and adhesion molecules causes a chemotactic gradient that promotes the influx of leukocytes to the kidney (Vergoulas et al., 2009). Infiltration of macrophages and neutrophils has been reported 6 h after BD in rat (Takada et al., 1998). In the same context, granulocytes infiltrate the renal cortex immediately after experimental onset of BD in rats and peak 2 h after induction of injury (Schuurs et al., 2006). This neutrophil infiltration into the organ pre-transplantation can exacerbate tissue damage and compromise graft function in the recipient.

Within the context of inflammation and leukocyte recruitment, the endothelium, particularly its extracellular matrix known as the endothelial glycocalyx (EG), may also play a role. The endothelium is a dynamic and multifunctional structure involved in vasomotor regulation, maintaining vascular homeostasis, mediating immune response through leucocytes recruitment and diapedesis, and regulating various other physiological processes such as coagulation and angiogenesis (Rajendran et al., 2013; Pillinger and Kam, 2017). At the heart of these functions, the, EG, a carbohydrate-rich, negatively charged hair-like layer covering the luminal surface of endothelial cells (EC), emerges as a key structure. Composed of proteoglycans, glycosaminoglycans, glycoproteins, and glycolipids (Jedlicka et al., 2020), the, EG acts as a protective barrier and regulator of the endothelial response (Jourde-Chiche et al., 2019): it contributes to endothelial functions by regulating vascular permeability, mediating leukocyte diapedesis, facilitating endothelial repair, modulating angiogenesis, and participating in mechanotransduction (Filippi, 2016; Song et al., 2018; Yilmaz et al., 2019).

In many physiological and pathophysiological stresses such as disturbed blood flow, diabetes mellitus, sepsis, acute and chronic kidney diseases, and ischemia/reperfusion injury (Rajendran et al., 2013; Lopez-Quintero et al., 2013; Ostrowski et al., 2015; Jan-Sören et al., 2014), the glycocalyx undergoes deleterious changes affecting its protective role, initiating endothelial activation, and subsequently leading to endothelial dysfunction within vital organs such as the heart, lungs, liver, and at the core of this study, the kidneys (Zhang et al., 2018; Yilmaz et al., 2019). Endothelial dysfunction and glycocalyx alterations are known to perpetuate and amplify each other. Oxidative stress and ROS generated during inflammation contribute to further damage to the glycocalyx, worsening endothelial dysfunction. This process forms a self-sustaining and detrimental feedback loop, often referred to as a “vicious circle” (Zhang et al., 2018; Ali et al., 2019).

While glycocalyx degradation is well described in the aforementioned pathological conditions, what happens to its structure during BD remain largely unexplored., though inflammation and oxidative stress generated during BD are recognized as significant factors that could contribute to glycocalyx damage (Zou et al., 2021).

Considering the large demand in kidney transplantation, the shortage of available organ, the necessity to expand organ selection criteria, and the exacerbating impact of BD on organ quality, it becomes imperative to preserve the graft through therapeutic interventions in the donor. Studies suggest that immunosuppressive agents such as tacrolimus (FK506), may have protective potential for long-term kidney transplant outcomes (Yuksel et al., 2019; Banas et al., 2020). Using a large-animal model, Belhaj et al. previously reported that pretreatment of BD pigs with FK506 not only prevented pulmonary capillary hypertension, but also right ventricle dysfunction induced by BD, through the inhibition of apoptosis and decrease of inflammation (Belhaj et al., 2022; Belhaj et al., 2023).

In this study using the same animal model of BD, we aimed to assess the impact of BD on apoptotic, inflammatory, and endothelial response processes in the kidney. Additionally, we focused on evaluating glycocalyx alteration, a key element in limiting inflammatory cell infiltration. Drawing upon previous research on the effects of BD on myocardial and pulmonary function, as well as the known benefits of FK506 (Belhaj et al., 2022; Belhaj et al., 2023), we hypothesized that this calcineurin inhibitor would alleviate renal alterations by protecting the endothelium through graft preconditioning after BD-induced injury in a porcine model.

## 2 Materials and methods

The current study received approval from the Institutional Ethics Committee on Animal Welfare at the Faculty of Medicine of the Université Libre de Bruxelles (protocol number: 510N).

### 2.1 Animal model of brain death

BD was induced as previously described (Belhaj et al., 2017; Belhaj et al. 2022; Belhaj et al. 2023). Briefly, the study included twenty-four 4-month-old female pigs (mean weight, 50 ± 4 kg; range 43–56), randomized to nine placebo-pretreated (brain death, BD) and eight FK506 pretreated pigs with brain death (BD + FK506) and seven served as controls (ctrl). The pigs used in this study were female because our animal facility is not equipped to manage the more aggressive and difficult-to-handle males. We acknowledge that this is a limitation of our study.

**FIGURE 1:**
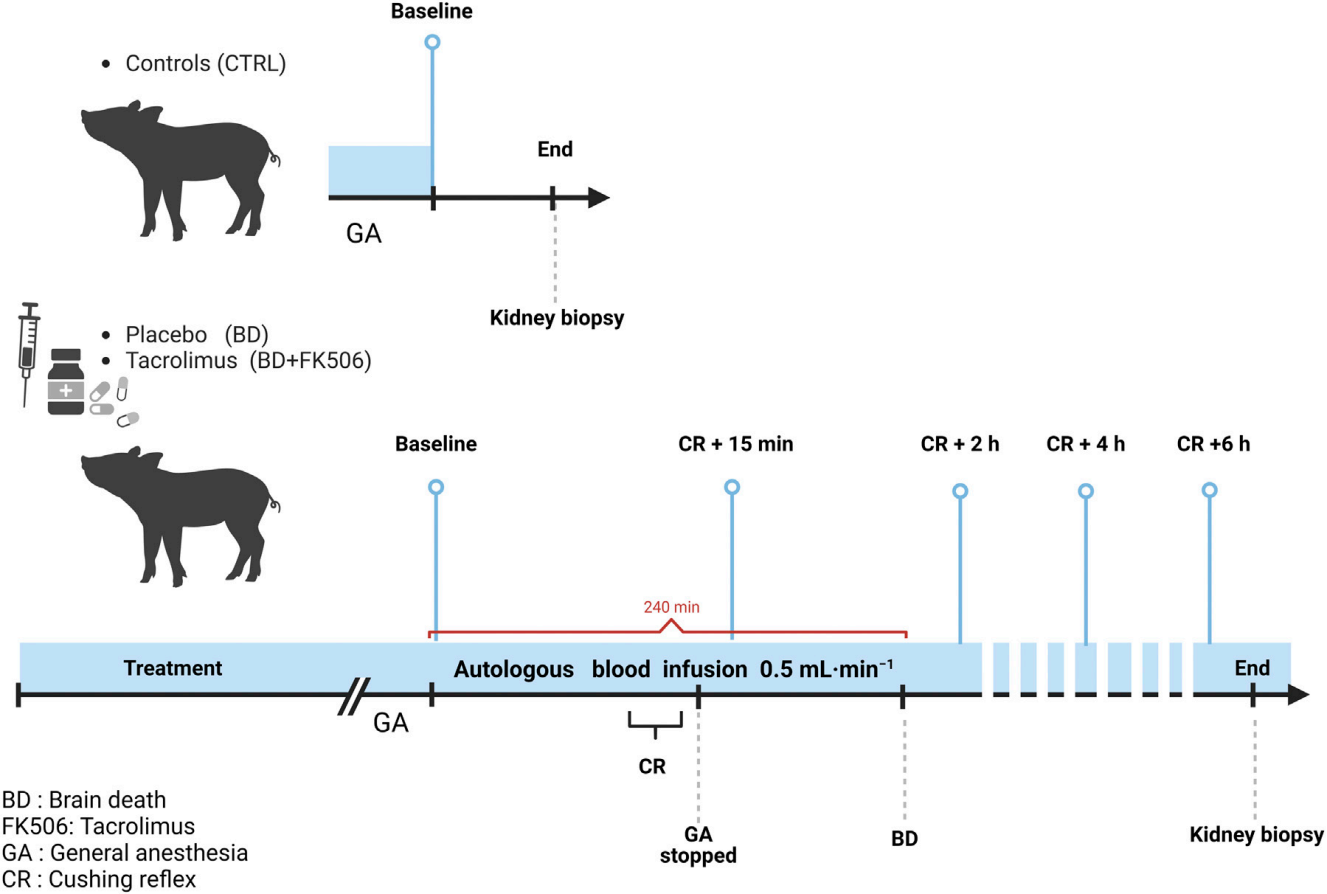
Schematic representation of the protocol timeline for controls (Ctrl, n = 7), placebo-pretreated (BD, n = 9) and tacrolimus-pretreated (BD + FK506, n = 8) brain death groups. Light blue lines indicate the time points for data set (hemodynamic measures, blood gases), urine, and blood sample collection. Created with BioRender.com.

Pretreatment with FK506 (PROGRAF^®^; Astellas, Japan; in lactose monohydrate) consisted in 0.25 mg kg^−1^ orally administered twice the day before the procedure followed by intravenously 0.05 mg.kg^−1^ 1 h before BD induction. After premedication and anesthesia, BD was induced by trepanning and gradually infusing autologous blood into the parenchyma of the temporoparietal cranium, at a rate of 0.5 mL per min over a period of 240 min. Administration of drugs used for anesthesia and paralysis was stopped when the Cushing reflex (CR), i.e., the neurophysiological response to abrupt increase in intracranial pressure (ICP) that is characterized by the manifestation of Cushing’s triad (bradycardia, increased blood pressure and irregular breathing patterns) was observed (Dinallo and Waseem, 2023). As previously reported, balanced crystalloid- and gelatin-modified solutions were perfused to maintain a right atrial pressure between 6- and 8-mmHg, at a rate of 15 ± 1 vs 13 ± 1 mL⋅kg−1⋅h−1 in the BD and BD + FK506 groups, respectively (*p* > 0.05). Additionally, if systemic arterial pressure fell below 65 mm Hg, a noradrenaline infusion was initiated and modulated to ensure adequate organ perfusion (Belhaj et al., 2022). One h after the end of CR, which lasted between 30 and 60 min, BD was objectivated by the standard clinical procedures (Morenski et al., 2003), i.e., i) evidence of profound coma, ruling out reversible factors like anesthesia or hypothermia; ii) absence of ocular papillary and corneal reflex on two occasions; iii) lack of spontaneous ventilation during the apnea test; and iv) no acceleration of heart rate following the administration of 1 mg atropine over 5–15 min. Six h following onset of CR the animals were euthanized by barbiturate overdose (Nembutal^®^, Oak, United States). For the ctrl group, animals were euthanized after the first data collection (immediately after all preparations and just before the start of the brain blood infusion, baseline = T0). The experimental design is shown in Figure 1.

### 2.2 Samplings

Urine and blood samples were collected at baseline (T0), 15 min (CR +15 min), two (CR + 2 h), four (CR + 4 h), and 6 h (CR + 6 h) following onset of CR.

Blood samples were collected into ethylenediaminetetraacetic acid (EDTA) anticoagulant tubes. They were then immediately centrifuged, and the serum was collected and stored at −80°C. Urine samples were collected directly from the bladder using a urinary catheter and a collection bag equipped for hourly diuresis measurement. Samples were stored at −80°C until analysis.

Kidney tissue samples were collected at (CR+ 6 h). A part (5–7 cm long) of the organ (cortex and medulla) was immediately immersed in liquid nitrogen and stored at −80°C for biological analysis, while the other part was preserved in formaldehyde 4% for 24 h, then paraffin-embedded for histological analysis.

### 2.3 Histological analysis

#### 2.3.1 Periodic acid schiff (PAS) & hematoxylin-eosin-saffron (HES)

Paraffin-embedded kidney sections (3-μm thickness) underwent staining with Periodic Acid Schiff (PAS) and hematoxylin-eosin-saffron (HES) staining to evaluate morphological changes. In our study, 6 images per animal were evaluated for the size of glomeruli using Image J, while discoloration of nuclei, alteration of brush border, necrotic tubules, and tubular casts were assessed through visual observation in a blinded manner. Data and representative images are included in the Supplementary Material.

### 2.4 Biological parameters

#### 2.4.1 Osmolality measurement

Osmolality of urine samples was measured using the freezing point depression method (Osmo1, Advanced Instruments Inc., Norwood, MA, United States). Osmolality values were expressed as mOsm/kg H2O and represent the mean value of two separate measurements. The coefficient of variation (CV) was under 0.5%.

#### 2.4.2 Urine protein-creatinine ratio–UPCR

Protein concentration was assessed using the Pierce BCA protein assay kit (Cat. #: 23225; Thermo Scientific, Rockford, United States). Optical density was measured at λmax 562 nm. Results for each sample represented the mean value of two separate measurements. The BCA assay detects protein concentrations from 20 to 2,000 μg/mL, the CV is 14.7%.

Urine creatinine level was determined based on the Jaffe principle of alkaline creatinine-picric acid complex color formation. Optical densities were measured at λmax 520 nm using a UV–visible spectrophotometer. All samples were run in duplicate. The detection limit ranged from 0.21 to 37.85 mg/dL, with a coefficient of variation (CV) of less than 2.3%.

The urine protein-creatinine ratio (mg/mg) represents the level of proteinuria (Wu et al., 2012).

### 2.5 Immunohistochemistry

#### 2.5.1 Terminal deoxynucleotidyl transferase dUTP nick-end labeling–TUNEL

Paraffin-embedded kidney sections (3-μm thickness) were used to detect kidney cells undergoing apoptosis through TUNEL staining using the ApopTag^®^ plus *in situ* peroxidase apoptosis detection kit (Merck Millipore, Temecula, CA, United States), as previously described (Mohan et al., 2015). Apoptotic nuclei were colored in brown using colorimetric substrates diaminobenzidine (DAB), while a counterstaining (HE) was applied to distinguish the remaining nuclei in blue.

The relative intensity of apoptosis was quantified using ImageJ software (v1.54d, National Institutes of Health, https://imagej.net/ij/). The rate of apoptosis within each structure (glomeruli, tubules, and vessels) was determined as the ratio of TUNEL-positive staining (brown nuclei) to total nuclei staining (brown + blue nuclei) expressed as a percentage (x100). For each structure, twenty fields per kidney were captured using light microscopy (Leica DM750). Apoptosis quantification was carried out by two operators blinded to the animals’ treatment.

#### 2.5.2 Myeloperoxidase (MPO) staining

Immunohistochemistry for myeloperoxidase (MPO) was performed to identify Myeloperoxidase Positive Inflammatory Cell Infiltrate in pig kidneys. Formalin-fixed paraffin-embedded (FFPE) 3-μm thick sections were submitted to immunohistochemistry. Briefly, kidney sections were deparaffinized, rehydrated, then rinsed and pre-incubated with sodium citrate buffer (10 mM, pH 6.0) for antigen retrieval. Incubation with the primary antibody, polyclonal rabbit anti-human myeloperoxidase (MPO; AB-9535, Abcam, Cambridge, United Kingdom; 1:100 diluted in PBS with 1% BSA), was performed overnight at 4°C following manufacturer’s instructions. Signal Stain Boost IHC Detection Reagent (#8114S, Cell Signaling Technology, Germany) was added for 30 min at room temperature. Revelation of the secondary antibody was highlighted by the enzymatic action of peroxidase and diaminobenzidine (DAB) as chromogen. The sections were counterstained with hematoxylin, dehydrated, and mounted.

Finally, 40 to 60 fields of view were randomly selected and photographed to manually count the number of MPO-positive cells in each section using light microscopy (Leica DM750) and digitized using a ICC50 W micro camera (400 × magnification). Within each field, intravascular inflammatory cells were excluded. The analysis was carried out by two independent researchers in a blinded manner.

### 2.6 Immunofluorescence

#### 2.6.1 Wheat germ agglutinin (WGA) and platelet cell adhesion molecule 1 (PECAM-1/CD31)

Kidney cryosections of 10 µm were put on SuperFrost slides and fixed at 4°C (70% methanol, 5% glacial acetic acid, 3.7% formalin and 21.3% PBS 1X). Kidney sections were then incubated 1 h at room temperature in a blocking solution (10% TBS10X, 10% FBS, 3% BSA). Afterwards, immunofluorescence staining was performed using anti-wheat germ agglutinin (WGA), which binds to N-acetyl-D-glucosamine and N-acetyl neuraminic acid of GAGs, coupled with Alexa Fluor 488 (Invitrogen, W11261, 1/2,500 in PBS1X - BSA1%) for 30 min. A double immunostaining for EC markers was carried out using CD31 antibody (Abcam, ab28364; 1:50) at 4°C overnight, and an anti-rabbit coupled with Alexa Fluor 568 (Invitrogen, A11011, 1:200) for 1 h. This was followed by nuclei staining using Hoechst (33258 Sigma-Aldrich, 94403-1 ML); diluted (1:200) in PBS-BSA 3% for 30 min at room temperature. Coverslips were mounted with Mowiol solution.

Fifteen vessels (40–140 µm diameter) per section (animal) in cortex-medullary area were identified and visualized with a confocal microscope (Leica TCS-SP5, DM6000-CFS) at 40× magnification. Luminal WGA fluorescence intensity, delineated as the region of interest using CD31 labeling to outline the endothelium, was quantified using ImageJ software.

### 2.7 Statistical analysis

Statistical analyses were conducted using GraphPad Prism5 software (GraphPad Software Inc., San Diego, CA.). Prior to conducting statistical analyses, normality of distribution was assessed. All data were expressed as means ± SEM. For biological variables, the data were analyzed using a two-factor (group × time) repeated measures ANOVA (BD, BD + FK506, 5 times and interaction). For kidney tissues, group comparisons (Ctrl, BD, BD + FK506) were performed using One-way ANOVA, unless the distribution within a group deviated from normal (nonparametric), in which case the Kruskal–Wallis test was applied. A *p*-value below 0.05 was considered as statistically significant.

## 3 Results

### 3.1 Histological analysis of the kidney using HES and PAS staining

Upon examining the kidney sections with different staining techniques, no morphological alterations or notable changes were observed in the HES-stained kidney sections in both BD and BD + FK506 groups. Similarly, PAS staining did not reveal any abnormalities in the tissue architecture (Supplementary Figure S1). In summary, glomerular size remained unchanged across all three groups (Ctrl, BD, BD + FK506). Additionally, we observed no nuclei discoloration, alteration of brush border, necrotic tubules, or tubular casts in any of the three groups, indicating the absence of these features.

### 3.2 Renal function assessment through urine parameters

**FIGURE 2:**
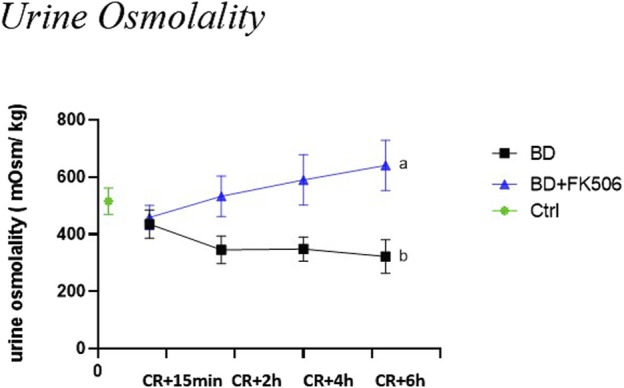
Urine osmolality evolution following brain death induction in pigs, investigation of the effects of pretreatment with tacrolimus. Urine osmolality (in mOsm/kg) at 15 min, 2 h, 4 h and 6 h after the Cushing reflex in controls (Ctrl; n = 7; green symbol), placebo-pretreated (BD, n = 9; black) and tacrolimus-pretreated (BD + FK506, n = 8; blue) brain death groups. The values are represented as mean (symbols) and standard error of the mean (bars), Curves with different letters significantly differ, (2-way repeated measures ANOVA, *p*-value <0.05).

At the onset of the experiment (CR+15 min), both the BD and BD + FK506 groups exhibited similar levels of urine osmolality (Uosm). Subsequently, urine osmolality was significantly lower in the untreated BD group than in the BD + FK506 group. The time-course of Uosm from CR to CR+6 h showed a decrease in the BD group and an increase in the BD + FK506 group (refer to Figure 2).

**FIGURE 3:**
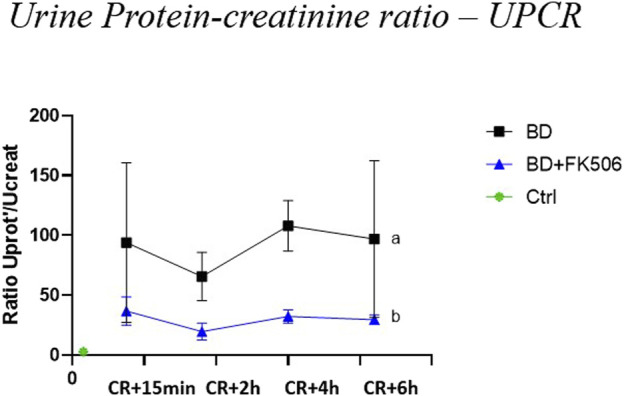
Evolution of urine protein-creatinine ratio –(UPCR) in brain dead pigs, following brain death induction, focus on the effects of pretreatment with tacrolimus. Protein-creatinine ratio –(UPCR) was evaluated at 15 min (CR + 15 min), 2 h (CR + 2 h), 4 h (CR + 4 h), and 6 h (CR + 6 h) after Cushing reflex (CR) in controls (Ctrl; n = 7; green symbol), placebo (brain death, BD; n = 9; black symbol), and tacrolimus-pretreated brain death (BD + FK506; n = 8; blue symbol) groups. The values are represented as mean (symbols) and standard error of the mean (bars). Curves with different letters significantly differ (2-way repeated measures ANOVA, *p*-value <0.05)

Baseline values (t0), being indicative of normal UPCR levels, showed no statistically significant difference between BD, BD + FK506 when compared to ctrls (Data not shown). However, subsequent analysis during BD progress revealed a significant increase in UPCR over time in the BD group compared to the FK506 pretreated group (Figure 3).

### 3.3 Apoptosis and inflammation as markers of renal injury

**FIGURE 4:**
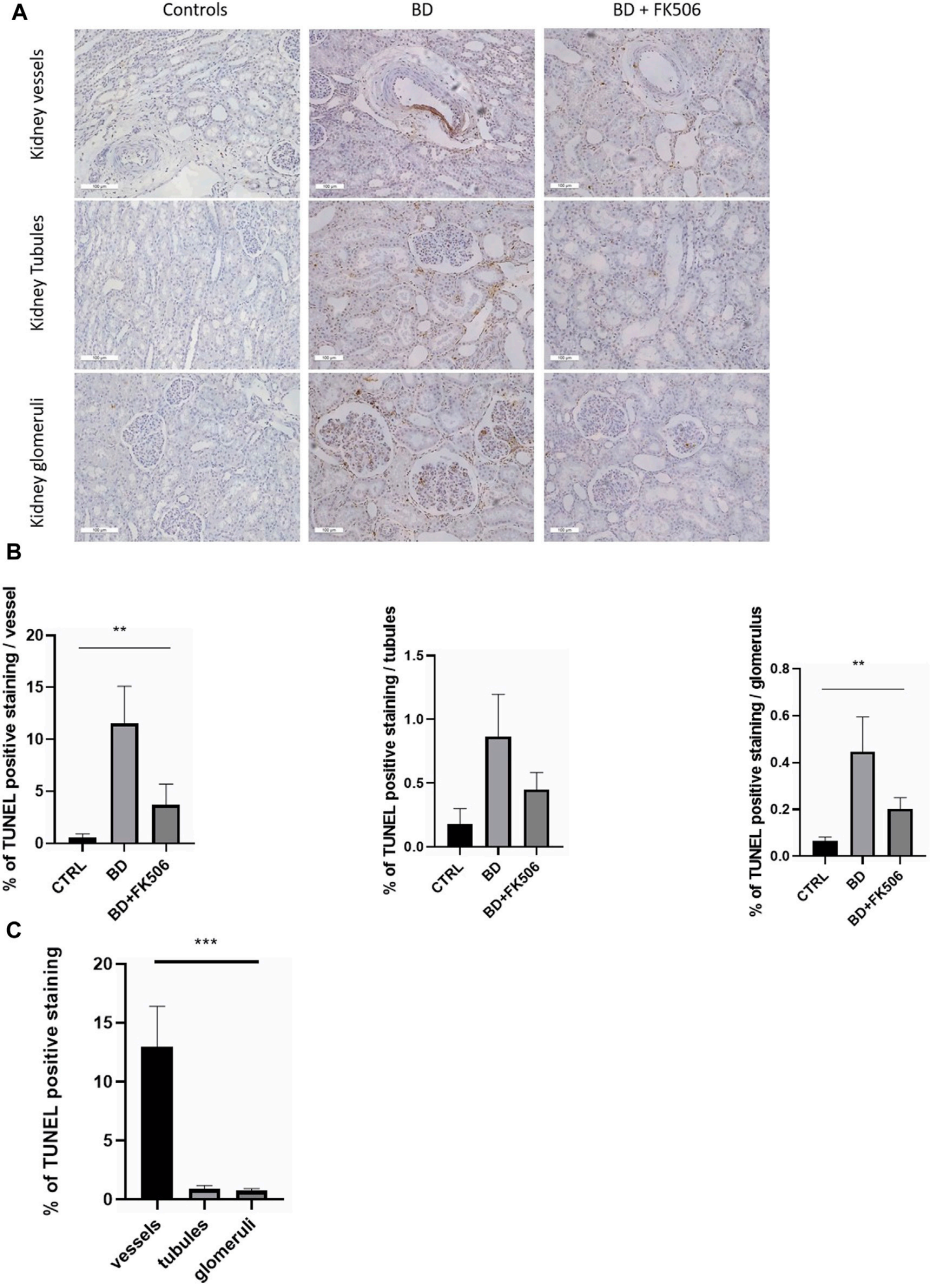
Evaluation of cell apoptosis in pigs with brain death, pretreated or not with tacrolimus. **(A)** Representative images of TUNEL staining in the kidney vessels, tubules and glomeruli are shown for each group (Controls (CTRL), brain death (BD), and tacrolimus-pretreated (BD + FK506) pigs). TUNEL-positive nuclei are stained in brown (DAB), and normal nuclei are stained in blue (HE). **(B)** Quantification of the percentage of apoptotic nuclei to total nuclei (healthy + apoptotic) in the kidney vessels, tubules and glomeruli (n = 20 images/structure per animal) of Controls (n = 5), BD (n = 9), and BD + FK506 (n = 8), measured using ImageJ. **(C)** Comparison of TUNEL-positive cells among three kidney structures: vessels, tubules, and glomeruli in the brain death group. Results are expressed as mean ± SEM. Kruskal–Wallis test **p ≤ 0.01, ***p ≤ 0.001.

To assess apoptosis within key kidney structures—glomeruli, tubules, and vessels—the TUNEL assay was performed, with representative images depicted in (Figure 4A). Kruskal–Wallis test revealed a significantly different amount of apoptosis among the three groups (Ctrl, BD, and BD + FK506) within glomeruli and vessels, whereas no significant differences were observed in tubules. BD induced a significant increase in kidney apoptosis in vessels and glomeruli compared to the controls. Tacrolimus treatment decreased apoptosis levels, showing less apoptosis than the BD group (Figure 4B). In both BD and BD + FK506 groups, apoptotic nuclei were predominantly localized within vascular walls, while a lower proportion was observed in the proximal and distal tubules, as well as in glomeruli (Figure 4C). Conversely, in ctrl group, apoptotic cells were rarely observed within all three structures.

**FIGURE 5:**
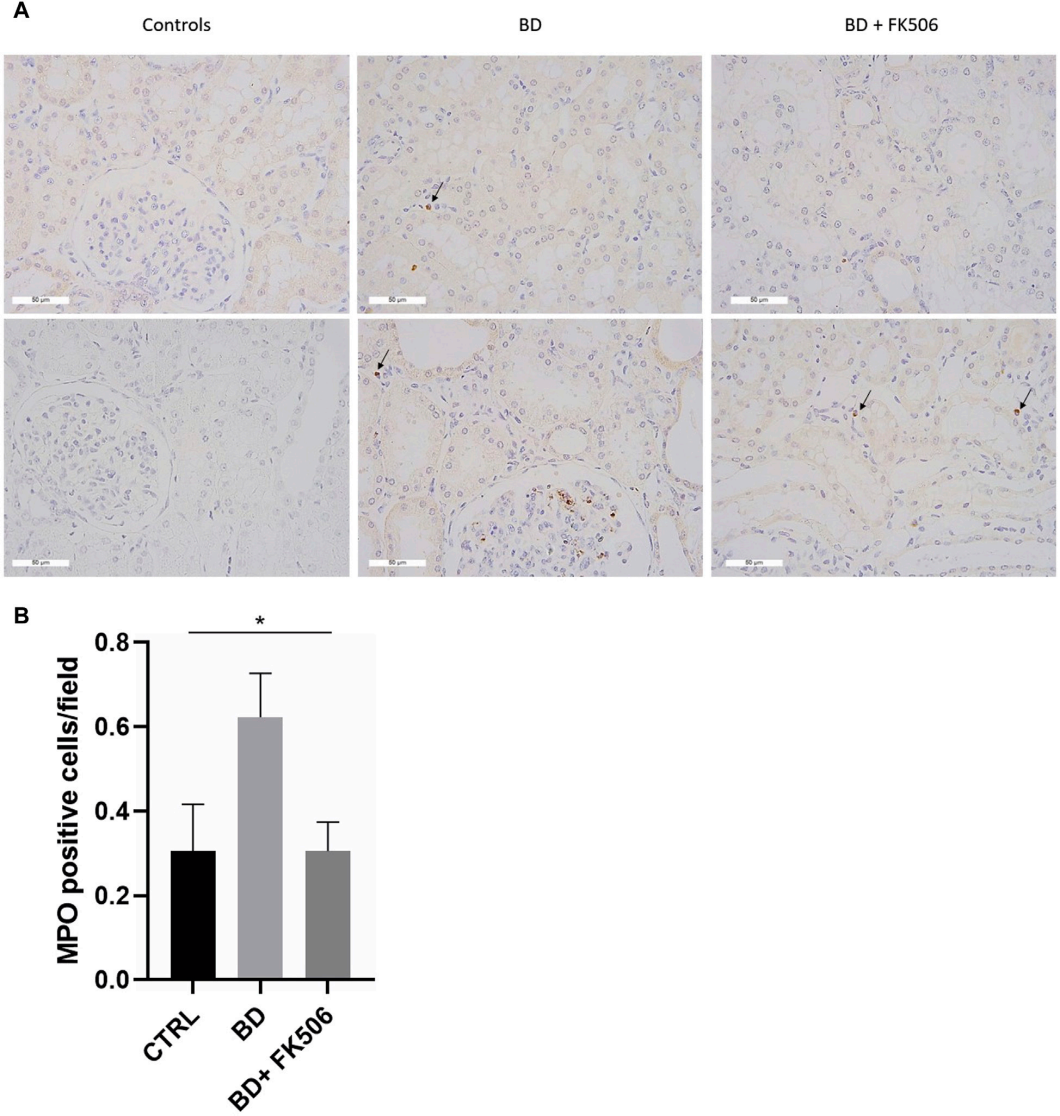
Immunohistochemistry analyses of myeloperoxidase-positive stained cells. **(A)** Representative images of immunohistochemistry for the neutrophil marker myeloperoxidase within the kidney tissue for each group: Controls (CTRL), brain death (BD), and tacrolimus-pretreated (BD + FK506) pigs. Myeloperoxidase (MPO) staining is indicated by brown staining (black arrows) at a magnification of ×400. **(B)** Quantification of neutrophil infiltration in the kidneys: counting of the average number of MPO-positive cells in CTRL (n = 5), BD (n = 9), and BD + FK506 (n = 8) groups; n = 40 to 60 fields of view. Results are presented as mean ± SEM, statistical analysis was conducted using one-way ANOVA. *p ≤ 0.05.

Kidney-infiltrating neutrophils were investigated, and the results are illustrated in Figure 5A. One-way ANOVA was conducted to compare neutrophil infiltration among the BD, BD + FK506, and ctrl groups (Figure 5B). The analysis revealed a significant difference among the groups (*, *p* < 0.05). Specifically, neutrophil infiltration was higher in the BD group compared to the ctrl group. Tacrolimus treatment exerted a protective effect against neutrophil infiltration, bringing it to levels almost identical to the ctrl group.

### 3.4 Endothelial glycocalyx (EG) degradation

As previously observed in our animal model, BD led to higher levels of circulating hyaluronic acid (HA) and Heparan Sulfate (HS) in the bloodstream. These markers of glycocalyx shedding decreased in the BD + FK506 group; suggesting that FK506 pretreatment prevented, EG deterioration (Belhaj et al., 2022).

**FIGURE 6:**
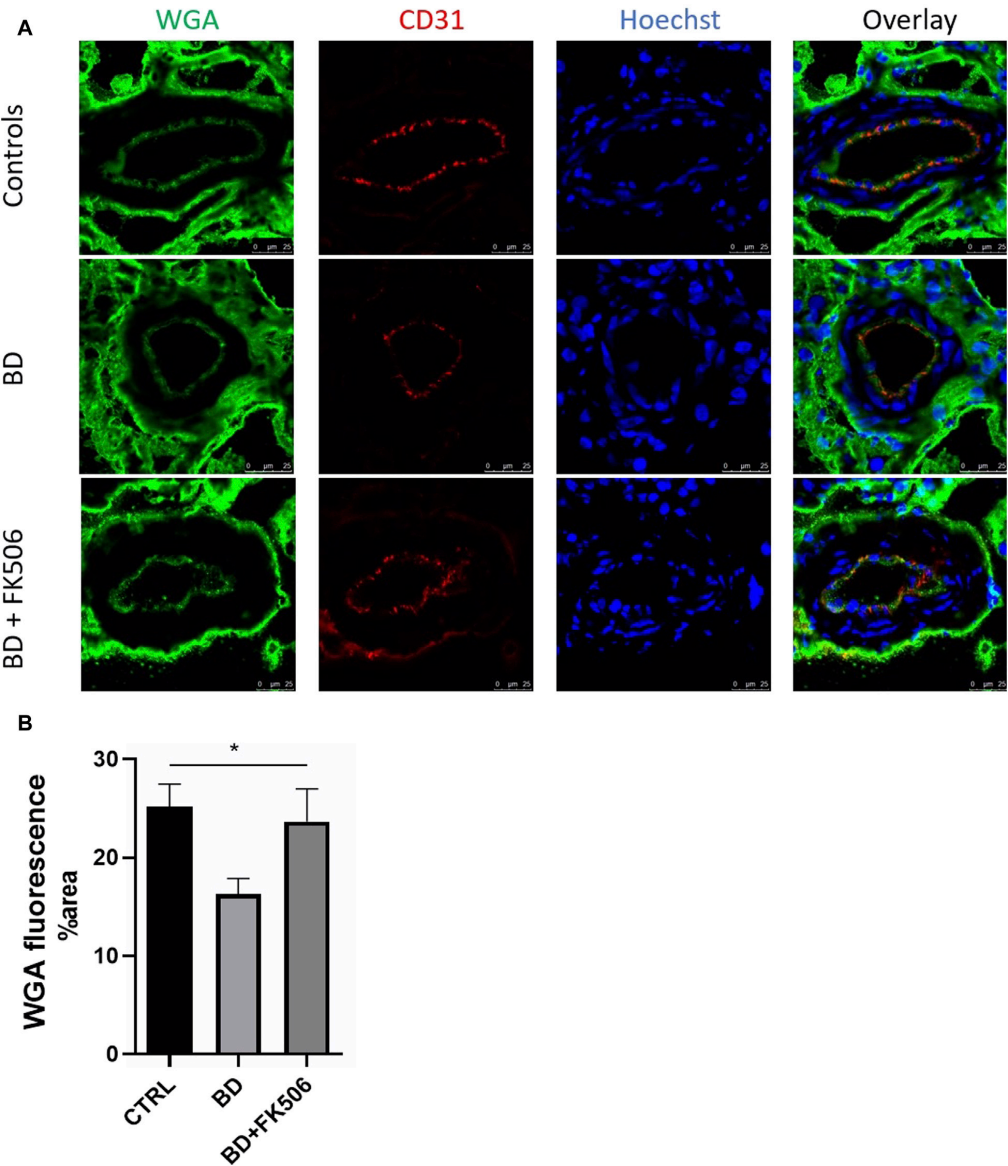
Quantitative analysis of glycocalyx in controls and brain-dead pigs with and without tacrolimus pretreatment. **(A)** Representative complementary wheat germ agglutinin (WGA) and endothelial cells marker PECAM1 (CD31) fluorescent labelling in kidney tissue using confocal microscopy. Glycocalyx in green, endothelial cells in red (CD31) nucleus in blue (Hoescht). Scale bar = 25 μm (magnification×400). **(B)** Quantification of WGA fluorescence signal intensity in each group: Control (CTRL), brain death (BD), tacrolimus-pretreated brain death (BD + FK506). n = 15 images per animal in each group. Results are represented as means ± SEM, One-way ANOVA *, *p* ≤ 0.05.

In this study, we used confocal microscopy to investigate damage to the glycocalyx, regulator of diapedesis and a key element in the initiation of endothelial dysfunction. One-way ANOVA analysis unveiled a statistically significant difference among the three groups (Ctrl, BD, and BD + FK506). Our analysis revealed a reduced green signal in BD kidney sections, indicative of glycocalyx degradation, compared to the ctrl group. Furthermore, the BD + FK506 group showed a green signal similar to that of the control kidney sections, suggesting that tacrolimus treatment protected against glycocalyx degradation (Figure 6).

## 4 Discussion

Our primary objective in this study was to examine the impact of brain death (BD) on renal physiology and to validate a sequence of events in a pig model. Following BD, we observed no structural damage but noted significant neutrophil infiltration and apoptosis, an increase in the proteinuria, signs of a loss in urinary concentration capacity, and damage to the glycocalyx. Our secondary objective was to evaluate the therapeutic effect of FK506. Brain dead pretreated pigs showed a reduction in UPCR, improvement in urinary concentration capacity, less neutrophil infiltration and apoptosis, and preservation of the glycocalyx. These findings highlight the protective effects of tacrolimus in mitigating renal damage and preserving kidney function following brain death.

### 4.1 Impact of brain death on renal function

After brain death (BD), significant alterations in renal function have been reported, including decrease in glomerular filtration (Nijboer et al., 2010) generally attributed to mechanisms such as sympathetic nervous system activation, hormonal disturbances, systemic inflammation, and alterations in renal perfusion (Bos et al., 2007; Armstrong-Jr et al., 2023). In our model, it is important to note that hypoperfusion did not occur, as the animals were continuously perfused large quantities of balanced crystalloid- and gelatin-modified solutions throughout the entire experimental process to maintain hemodynamic stability.

Our histopathological analysis of kidney tissue revealed no significant structural alterations. However, to gain a deeper understanding of the overall health of the tubular function and filtration barrier, we examined whether renal function was affected within the first 15 min to 6 h following CR. For this purpose, osmolality was used to assess the kidney’s ability to concentrate or dilute urine. The urine osmolality observed in BD and BD + FK506 groups fell within the typical broad range considered normal for pigs (Kaneko et al., 2008). A compelling observation emerges when considering the impact of FK506 pretreatment, as the pretreated group exhibited an increase in urine osmolality compared to the BD group which showed a decrease over time. BD is recognized to typically induce dysfunction within the hypothalamic-pituitary system, potentially impairing the synthesis and secretion of hormones, including the antidiuretic hormone (ADH) (Howlett et al., 1989). Consequently, the observed decline in urine osmolality over time in the BD group may be linked to compromised ADH production or release unlike the FK506 pretreated group where an increase is observed, suggesting a potentially different hormonal response. Indeed, a case report suggests that after liver transplantation, FK506 could augment renal sensitivity to ADH and induce its release (Takagi et al., 2017).

Subsequently, kidney glomerular damage was analyzed through proteinuria estimation using the UPCR. In this study, the UPCR became abnormal 15 min after the onset of the Cushing reflex. Our results showed significantly higher UPCR values over time in the BD group compared to the BD + FK506 group. Remarkably, FK506 pretreatment led to a 2-fold decrease in the severity of proteinuria compared to the BD group, highlighting its potential efficacy in limiting damage to the glomerular filtration barrier through its known anti-inflammatory effects. Overall, our findings indicate noticeable harm to renal function, characterized by proteinuria following BD. This corroborates previous research showing progressive impairment of kidney function and worsened proteinuria over time in BD donor groups compared to living donors (Francuski et al., 2009). However, FK506 pretreatment emerges as a significant factor in effectively attenuating this deterioration.

### 4.2 Apoptosis and inflammation in the kidneys of brain-dead pig

In this context of renal damage and inflammation, we sought to delve deeper into the cellular events contributing to this injury. Specifically, our focus shifted to apoptosis, a process tightly linked to inflammation, and an early indicator of cellular injury and dysfunction. Given that studies in the context of acute kidney injury suggest that apoptotic endonuclease activation typically occurs within the initial 24-h window following renal injury (Davis and Ryan 1998; Lu et al., 2019; Thomas et al., 2022), our study was well-positioned to observe apoptosis within this timeframe. Indeed, apoptotic cells were detected 6 h after CR in glomeruli, tubules, and vessels. Based on previous findings in this BD pig model, this observation may be linked to oxidative stress, as evidenced by elevated expression levels of markers such as HO-1 and HIF-1α in lung tissue (Belhaj et al., 2017).

Together with systemic inflammatory environment, apoptosis is known to potentiate inflammatory responses: apoptotic ECs release apoptotic extracellular vesicles known as apoptotic bodies that favor the recruitment of inflammatory cells (Dieudé et al., 2015; Cardinal et al., 2018; Hernandez et al., 2023), acting as potent chemoattractants for neutrophils and release various “find-me” signals (Ramos and Rudolf, 2024). In turn, the inflammatory microenvironment can exacerbate apoptosis, forming a cyclic relationship between cell death and inflammation. Considering these insights we investigated neutrophil infiltration within the renal tissue. We observed a significant increase in neutrophils associated with BD compared to controls. In alignment with our results, a study demonstrated the presence of neutrophil infiltration in 53% of kidney allografts from cadaveric donors whereas living related donor allografts did not exhibit any detectable neutrophil infiltration (Koo et al., 1998). Our results are also consistent with earlier results in our experimental model demonstrating that BD induces the release of pro-inflammatory cytokines and chemokines, which lead to an upregulated neutrophil infiltration in both right and left ventricles after BD (Belhaj et al., 2023).

### 4.3 Glycocalyx damage following brain death

After investigating apoptosis and leukocyte infiltration, we turned our attention to the endothelium’s regulatory role in leukocyte diapedesis by examining the glycocalyx. Given the glycocalyx’s role in limiting leukocyte adhesion to the endothelium, we assessed its thickness.

Our results showed a significant compromised glycocalyx integrity in renal vessels indicating endothelial injury following BD when compared with controls. This aligns with increased shedding of glycocalyx components (HA and HS) in plasma previously reported in our model (Belhaj et al., 2022).

This glycocalyx alteration following BD can be related to BD-induced hemodynamic changes and catecholamine storm that contribute to oxidative stress in the renal microenvironment (Ueno, Zhi-Li, and Itoh, 2000; Van Erp et al., 2018). This is supported by increased oxidative stress molecules (HO-1 and HIF-1α) in the lung tissue post-BD in our experimental model (Belhaj et al., 2017). Indeed, ROS exacerbate endothelial dysfunction and glycocalyx damage in many pathologies and through various pathways (N. Xu et al., 2020); both by directly oxidizing glycocalyx components leading to their breakdown, and indirectly by acting as signaling molecules, activating sheddases such as matrix metalloproteinases or heparanases. These enzymes, released by leukocytes and ECs themselves, cleave proteoglycans and glycosaminoglycans (Dogné and Bruno 2020; Jackson-Weaver et al., 2019).

Cytokines released following BD, such as TNF- α are reported to increase EC permeability and potentially glycocalyx degradation via heparanase activation (Xu et al., 2015). Several studies have demonstrated that pro-inflammatory cytokines induce a persistent EC activation, resulting in the increased expression of adhesion molecules such as VCAM-1 and ICAM-1 (Pober and Sessa, 2007; Robinson et al., 2021; Ryan et al., 2022). Activated ECs then promote firm adhesion and the trans-endothelial migration of leukocytes, predominantly neutrophils, into the vascular wall. In this process, the glycocalyx limits access of leukocytes to the endothelium, and ensures fine control of leukocyte recruitment to sites of inflammation (Moore et al., 2021).

Both the catecholamine and cytokine storms are reported to converge to exacerbate endothelial activation, dysfunction, and glycocalyx degradation. This creates a loop wherein oxidative stress triggers glycocalyx degradation, facilitating leukocyte infiltration into the subendothelium. Subsequently loss of glycocalyx antioxidant properties further perpetuates this cycle (Bos et al., 2007). Accordingly, our research model highlights BD-induced hemodynamic changes, inflammatory cytokine release, oxidative stress, and increased vascular cell adhesion molecules (Belhaj et al., 2017; 2022; 2016). Future studies should investigate cytokine-activated sheddases.

### 4.4 Protective role of tacrolimus

Pretreatment with FK506 revealed promising outcomes. In addition to FK506 positive effect on renal function, we observed reductions in apoptosis, neutrophils infiltration, and degradation of endothelial glycocalyx compared to untreated pigs.

The specific mechanism behind this protection could involve a suppression of cytokines such as TNF-α, a decrease in oxidative stress, and reduction in sheddase activity. Indeed, the calcineurin inhibitor FK506, is known to modulate immune responses by blocking nuclear translocation of the transcription factor-nuclear factor of activated T cells (NFAT), and subsequently, inhibiting the production of TNF-α and other pro-inflammatory cytokines (Chang et al., 2016; Su et al., 2023). By inhibiting the production of TNF-α, FK506 may prevent the upregulation of sheddases such as heparanase and thus ensure a preserved glycocalyx integrity, crucial for vascular health and regulation of leukocyte adhesion and infiltration (S. Chen et al., 2017).

This protective role is contradictory with FK506 nephrotoxicity, associated with high repeated dosages and long-term treatment in recipients. In this case FK506 nephrotoxicity predominantly affects several key components within the kidney, namely, vascular ECs, tubular epithelial cells, arteriolar myocytes, and interstitial fibroblasts. This is evidenced by vasoconstriction in both afferent and efferent glomerular arterioles induced by FK506, resulting in reduced renal blood flow and glomerular filtration rate (GFR), along with significant impairment of EC function. Additionally, changes in intrarenal hemodynamics and a decline in GFR were observed after treatment in rats (Bentata, 2020). Despite previous concerns regarding potential nephrotoxicity associated with high dosage of FK506 and long-term treatment, our findings reveal a promising therapeutic role for FK506 as a pretreatment (El-Reshaid et al., 2022; Terzi and Mustafa, 2022). Furthermore, Belhaj et al. reported less need for noradrenaline administration after FK506 pretreatment in the same model, adding another beneficial effect to FK506 (Belhaj et al., 2023). Indeed, the dose of vasopressors administered to the donor before harvesting, is directly linked to the delayed graft function of the kidney recipient. Kidney transplant recipients from donors receiving an increased phenylephrine dose were nearly seven times more likely to develop delayed graft function (Swanson et al., 2020).

Our study provides comprehensive insights into the impact of BD on renal physiology, demonstrating increased proteinuria, loss of urinary concentration capacity, apoptosis, neutrophil infiltration, and notably, for the first time, identified impact on the endothelial glycocalyx. Remarkably, FK506 pretreatment showed a robust protective effect against these pathological events. Given the scarcity of available transplants relative to demand, there is an urgent need to explore innovative approaches to optimize graft outcomes. Our findings underscore the therapeutic potential of FK506 in preconditioning grafts, providing a promising approach for mitigating the detrimental effects of BD on renal function and endothelial health. Notably, the potential of tacrolimus to preserve the glycocalyx is of particular interest, as the protection of the glycocalyx is increasingly identified as a promising therapeutic target in a range of pathological conditions. This translational research could significantly improve transplant outcomes, addressing the urgent need to optimize graft survival and function, ultimately paving the way for better patient care and more successful transplants.

## Data Availability

The original contributions presented in the study are included in the article/Supplementary Material, further inquiries can be directed to the corresponding author.
