# Supplementary material for: Cascading renal injury after brain death: Unveiling glycocalyx alteration and the potential protective role of tacrolimus
Source: Front Cell Dev Biol. 2024 Aug 6;12:1449209. doi: 10.3389/fcell.2024.1449209 (PMC11333349; doi:10.3389/fcell.2024.1449209)
Supplement: Supplementary file 1 [file Table1.DOCX]

Supplementary Material

# Supplementary Figures


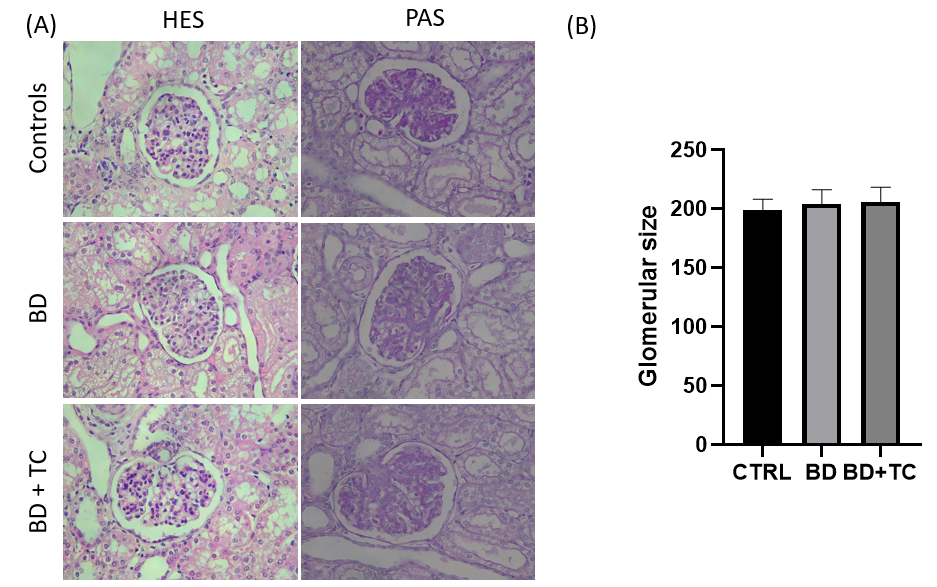


**Supplementary Figure 1.** Histopathological analysis. (A) Representative images of kidney sections stained with HES and PAS from the control (CTRL), brain death (BD), and brain death pretreated (BD+FK506) groups. (B) quantification of glomerular size in the three experimental groups: control (Ctrl), brain death (BD), and brain death pretreated (BD+FK506). Glomerular size was measured using digital image analysis with ImageJ from sections stained with HES. Data points represent the mean ± standard error of the mean (SEM).
